# Transcriptomics and non-targeted metabolomics reveal the mechanisms of leaf color changes in red-leaf cotton under drought stress and rewatering

**DOI:** 10.3389/fpls.2026.1766818

**Published:** 2026-02-16

**Authors:** Hu Zhang, Jinsheng Wang, Wenju Gao, Xiangyan Ma, Hongbin Wang, Wen Zhang, Qingtao Zeng, Jianping Li, Quanjia Chen, Qin Chen

**Affiliations:** 1 Xinjiang Key Laboratory of Crop Biology Breeding, College of Agriculture, Xinjiang Agricultural University, Urumqi, China; 2 National Key Laboratory of Cotton Bio-breeding and Integrated Utilization, Institute of Cotton Research of Chinese Academy of Agricultural Sciences (CAAS), Anyang, China; 3 The 7th Division of Agricultural Sciences Institute, Xinjiang Production and Construction Corps, Kuitun, China; 4 Cotton Research Institute of Xinjiang Uyghur Autonomous Region Academy of Agricultural Sciences (Xinjiang Cotton Technology Innovation Center/National Cotton Engineering Technology Research Center), Urumqi, Xinjiang, China

**Keywords:** drought stress, flavonoid biosynthesis, metabolomics, red-leaf cotton, transcriptomics

## Abstract

This study aims to investigate the pattern of dynamic leaf color changes (red-green-red) in red-leaf cotton under drought stress and rewatering, and to reveal the underlying molecular and biochemical mechanisms. Integrated transcriptomics and metabolomics analyses, combined with weighted gene co-expression network analysis (WGCNA), were employed to systematically study the physiological, gene expression, and metabolite changes in red-leaf cotton under mild drought, severe drought, and after rewatering. Under mild drought stress, red-leaf cotton accumulated higher levels of anthocyanins while maintaining relatively good photosynthetic performance, demonstrating an effective photoprotective response. In contrast, severe drought stress led to a significant decrease in anthocyanin content, accompanied by sharply reduced water retention and photosynthetic capacity, indicating a shift in physiological strategy towards survival priority. After rewatering, red-leaf cotton reactivated the flavonoid biosynthesis pathway, gradually restored anthocyanin synthesis, and showed clear phenotypic recovery. Transcriptomic analysis revealed the reprogramming of gene expression related to anthocyanin synthesis and drought tolerance pathways. Metabolomic analysis identified metabolites such as phenylalanine and 2-hydroxyquinoline, which provide precursors for anthocyanin synthesis.The research indicates that red-leaf cotton responds to drought and rewatering by dynamically regulating the flavonoid synthesis and metabolic network, demonstrating robust metabolic repair and stress memory capabilities. These mechanisms provide important theoretical support for breeding drought-resistant cotton varieties.

## 1 Introduction

Cotton (***Gossypium* spp.**) is one of the most important fiber and oil crops globally, contributing 35% of the world’s total fiber production and being widely cultivated in various climatic zones for fiber and oilseed production (Shuli et al., 2018). In fact, the cotton industry has been severely impacted by drought and high-temperature stress, with fiber yields declining by as much as 34% (Ul-Allah et al., 2021). Cotton reaches its peak water demand during the early boll development stage and as soil moisture gradually decreases, leading to water stress and reduced yields (Datta et al., 2019). Studies by Levi and others show that water stress can reduce cotton seed yield by 31% (Levi et al., 2009). Regardless of whether irrigation is used, cotton frequently faces drought stress, which lowers both yield and fiber quality. Against the backdrop of increasing global climate change and water shortages, the expansion of cotton cultivation into arid regions has made drought-resistant gene identification and the in-depth analysis of cotton’s molecular and physiological mechanisms of drought resistance a key focus of current agricultural research (Khan et al., 2023).

Red-leaf cotton, due to the presence and distribution of anthocyanins in the stems and leaves, exhibits a red coloration across the entire plant(N. Wang et al., 2022). This trait was first discovered by LEAKE in tree cotton and was referred to as red-leaf cotton (Tracy, 1896). To date, four classic genetic loci associated with anthocyanin accumulation have been identified in upland cotton: R1, R2, Rd, and Rs(X. Li et al., 2019). Among these, R1 and Rs control the appearance of red color in all tissues except cotton fiber (including red leaves, red stems, and red flowers). Natural anthocyanins have strong antioxidant and free radical scavenging properties, which make anthocyanin-rich cotton leaves highly resistant to ultraviolet radiation and intense light(D. W. Lee and Gould, 2002). Compared to green-leaf varieties, the red-leaf mutant of upland cotton (Rs) exhibits stronger photosynthetic efficiency under high light conditions (Shao et al., 2022). GhPAP1D encodes an R2R3-MYB transcription factor, and its overexpression leads to increased anthocyanin accumulation in transgenic tobacco and cotton. The overexpressing red-leaf cotton shows increased resistance to cotton bollworm, and red spiders that feed on red-leaf cotton exhibit reduced survival rates and fewer eggs compared to green-leaf varieties (Liang et al., 2020). Furthermore, Long and colleagues discovered that in spontaneous mutant cotton, the accumulation of flavonoids not only causes red coloration but also enhances resistance to *Verticillium dahliae* and *Botrytis cinerea* (Long et al., 2019). Liu and others showed that compared to non-anthocyanin tissues, anthocyanin-rich red pear fruits and purple pepper leaves exhibit more stable and higher photosynthetic capacity and light tolerance(Y. Liu et al., 2018).

The biosynthesis of anthocyanins begins with the phenylpropanoid pathway, with key rate-limiting enzymes such as phenylalanine ammonia-lyase (PAL), 4-coumaroyl-CoA ligase (4CL), and chalcone synthase (CHS) playing a critical role in flavonoid accumulation (Sharma et al., 2024), especially in stress responses like drought (Ma et al., 2014). Cinnamate-CoA, the first precursor for anthocyanin synthesis, is synthesized from phenylalanine through the catalytic actions of PAL, C4H, and 4CL (Ananga et al., 2013). The first step in the phenylpropanoid pathway is catalyzed by PAL, which deaminates phenylalanine to produce trans-cinnamic acid, linking primary and secondary metabolism in plants (Paul et al., 2023). In plants, the activity of 4CL is positively correlated with anthocyanin and flavonol content in response to stress, and PAL, C4H, and 4CL are usually co-expressed (Ma B. et al., 2024). Studies by Xiang and others suggest that PibH8 interacts with OsPAL1, the rate-limiting enzyme in the rice phenylpropanoid biosynthesis pathway. PibH8 protects OsPAL1 from degradation by competing with E3 ubiquitin ligase OsFBK16, enhancing PAL activity and increasing lignin and flavonoid content, thereby improving drought resistance in rice (Xiang et al., 2025). Xu and colleagues characterized the transcriptomes and metabolomes of drought-sensitive (D) and drought-resistant (XL) barley strains through a time-series design. The results indicated that the phenylpropanoid metabolic pathway was reprogrammed, downregulating the lignin metabolic pathway while enhancing flavonoid and anthocyanin biosynthesis, which improved drought resistance (Xu et al., 2021).

Transcriptomics refers to the total RNA (including mRNA, lncRNA, miRNA, etc.) transcribed from a specific cell or tissue under a certain state, reflecting the expression levels and regulatory status of genes comprehensively (Magar et al., 2022). Metabolomics is the collection of endogenous small molecules (such as sugars, amino acids, and organic acids) in organisms, directly reflecting the physiological status and biochemical activity of cells (Wishart, 2019). Integrated transcriptomics and metabolomics analysis can link gene expression with metabolite changes, constructing a complete story of “gene—enzyme—metabolite—phenotype” and enabling a more comprehensive and mechanistic interpretation of life phenomena (Lai et al., 2023).

This study focuses on the unique “red-green-red” dynamic leaf color change phenomenon exhibited by red-leaf cotton under drought stress and rewatering. This complete and reversible color change has not been systematically reported in any cotton resources, representing a novel drought response pattern. The phenomenon not only intuitively reflects the dynamic adaptation of red-leaf cotton to water stress but also suggests a systematic metabolic regulation mechanism. Although previous research has revealed the important role of anthocyanins in plant stress resistance (Naing and Kim, 2021), the mechanisms through which they dynamically accumulate and degrade in response to drought stress via a multi-layered regulatory network, particularly the “gene-metabolite-phenotype” cascade regulation pathway driving this complete reversible color change process, still lack a systematic analysis. Therefore, this study aims to integrate transcriptomics and metabolomics analyses to systematically explore the molecular and biochemical basis of red-leaf cotton’s dynamic leaf color changes, with the goal of revealing its physiological and ecological functions in drought adaptation and providing theoretical support for elucidating plant smart resistance strategies and breeding drought-resistant cotton varieties.

## 2 Material and methods

### 2.1 Planting and treatments

This study used the upland cotton variety “Red-leaf Cotton” as the experimental material, which was planted in 2025 in Huoyanghe City, Xinjiang Uygur Autonomous Region. The experiment employed a planting pattern of 1 membrane with 3 rows, and 1 row with 1 belt, with each row measuring 2 meters in length. Prior to the boll development stage, all plants received regular irrigation and natural precipitation management. At the boll development stage, drought stress treatment was applied by halting irrigation. Samples of the third-to-last leaf from each plant were collected on the 0th day (CK), the 5th day (LD), and the 10th day (SD) of the drought stress treatment, with 3 biological replicates per group. The samples were quickly wrapped in aluminum foil and frozen in liquid nitrogen, then stored at -80 °C for later analysis. After 10 days of sustained drought, rewatering was applied, and samples were collected again on the 7th day after rewatering (RW) following the same method. All samples were then sent to Guangzhou Gideo Biotechnology Co., Ltd. for transcriptome sequencing library construction and non-targeted metabolomics analysis.

### 2.2 Physiological index measurements

The anthocyanin content was determined using the pH differential method(J. Lee et al., 2005). A precise 0.1 g of the liquid nitrogen ground sample was weighed, and 1 mL of acidic ethanol extraction solution (concentrated hydrochloric acid: 80% ethanol = 3:97, v/v) was added. The sample was ultrasonically extracted for 30 minutes and then centrifuged to collect the supernatant. The absorbance was measured at the maximum absorption wavelength, 620 nm and 650 nm, using a microplate reader.

The corrected value was then calculated using the formula:


 ΔA=(Amax−A620)− 0.1×(A650 − A620)

The anthocyanin content was then calculated using the formula:


[(ΔA × V × 10^−3)/ (ϵ × d) × M1 × F]/(M × 10^6)

where V = 0.5 mL, ϵ = 2.69 × 10^4 L/mol/cm, d = 0.6 cm, M1 = 449.2 g/mol, F is the dilution factor, and M is the sample mass (g).

Chlorophyll content was measured according to the Lichtenthaler method (Lichtenthaler, 1987). A 0.1 g powder sample was added to 10 mL of 95% ethanol and extracted in the dark until the tissue turned white. Absorbance at 663 nm and 645 nm was measured.

The chlorophyll a content was then calculated using the formula:


(12.72 × A663 − 2.59 × A645)/M × V × F

The chlorophyll b content was then calculated using the formula:


(22.88 × A645−4.67 × A663)/M × V × F

The total chlorophyll content was then calculated using the formula:


Chlorophyll content = chlorophyll a+chlorophyll b

where M is the sample mass (g), V is the extraction volume (0.01 L), and F is the dilution factor before measurement.

Relative water content (RWC) was determined by weighing the fresh weight (FW) immediately after sample collection, followed by placing the samples in a 105 °C oven for 15–30 minutes to kill the tissue, and then drying them at 80 °C to a constant weight (24 hours) (Mullan and Pietragalla, 2012). After cooling the dried samples in a desiccator to room temperature, the dry weight (DW) was measured. The relative water content (RWC%) was then calculated using the formula:


RWC%=(FW −DW)/FW×100%

where FW is the fresh weight and DW is the dry weight.

Leaf chlorophyll content (SPAD) was measured using the SPAD-502Plus chlorophyll meter (Konica Minolta, Japan) for non-destructive detection (Süß et al., 2015). For each replicate, 10 healthy fully expanded leaves were selected, and three measurement points in the middle region of the leaf, avoiding the main vein, were measured. The average value of the individual leaves was calculated, and the final SPAD value for the replicate was the average of the 10 leaves.

Photosynthetic parameters were measured using a portable photosynthesis measurement system (CIRAS-3, PP Systems, UK) (Hornyák et al., 2024). The third-to-last leaf of the cotton plant was selected to measure the net photosynthetic rate (Pn), stomatal conductance (Gs), intercellular CO2 concentration (Ci), transpiration rate (Tr), water use efficiency (WUE), and vapor pressure deficit (VPD). Three plants were continuously measured for each material as one biological replicate, with three independent experimental repeats.

### 2.3 RNA extraction and transcriptome library construction

Total RNA was extracted using the TIANGEN Plant Total RNA Extraction Kit for polysaccharide and polyphenol-rich samples. All procedures were performed in an RNase-free environment. After tissue samples were ground in liquid nitrogen, they were subjected to lysis, organic solvent extraction, or column purification according to the corresponding methods. The final RNA was dissolved in RNase-Free Water and stored at -80 °C (Brown et al., 2018).RNA quality was systematically assessed using agarose gel electrophoresis, NanoDrop 2000 spectrophotometer, and Agilent 2100 Bioanalyzer. Electrophoresis was used to check the integrity of rRNA bands, NanoDrop was used to analyze the A260/A280 and A260/A230 ratios for purity assessment, and the Agilent 2100 Bioanalyzer was used to quantify RNA quality through the RNA Integrity Number (RIN).Transcriptome sequencing libraries were constructed using the Hieff NGS^®^ Ultima Dual-mode mRNA Library Prep Kit. The process included: Oligo(dT) magnetic beads were used to enrich mRNA, which was then fragmented; the fragmented mRNA served as a template to synthesize double-stranded cDNA; Illumina-compatible adapters were ligated; and finally, the library was amplified through PCR. The entire process was purified using Hieff NGS^®^ DNA Selection Beads. The resulting libraries were quality controlled using the Agilent High Sensitivity DNA or DNA 1000 Assay Kit on the Agilent 2100 system to ensure that the insert fragment size and concentration met the requirements for sequencing (Kumar et al., 2012).

### 2.4 Transcriptome gene expression quantification, differential analysis, and functional enrichment

Based on the upland cotton TM-1 reference genome (https://www.cottongen.org/species/Gossypium_hirsutum/CRI-TM1) (F. Li et al., 2015), the genome index was first constructed using HISAT2 v2.0.5, and clean reads were then aligned to the reference genome (Kim et al., 2019). Gene read counts were calculated using featureCounts (Liao et al., 2014), and gene expression levels were normalized based on FPKM values. Differential expression analysis was subsequently performed using DESeq2 software (Love et al., 2014) and edgeR software (Robinson et al., 2010) (P-values were adjusted using the Benjamini-Hochberg method, with a threshold set at adjusted P ≤ 0.05 and |log2FoldChange| ≥ 1). Finally, GO functional annotation and KEGG pathway enrichment analysis were conducted on the significantly differentially expressed genes using the clusterProfiler package(T. Wu et al., 2021).

### 2.5 WGCNA analysis

A weighted gene co-expression network was constructed using the WGCNA R package (Langfelder and Horvath, 2008)(v1.72-5) to systematically identify co-expressed gene modules associated with cotton’s physiological response to drought stress. First, based on the FPKM matrix of differentially expressed genes, a scale-free co-expression network was constructed using a soft-threshold power function (power = 14, determined by the scale-free topology fit index and average connectivity). Highly co-expressed genes were then grouped into different modules using the dynamic hybrid cutting method. Next, the correlation between the module eigengenes and the measured physiological phenotype data (including relative water content, chlorophyll content, etc.) was calculated to identify key modules significantly associated with specific drought-related physiological traits. Finally, gene-gene connectivity relationships were extracted from the key modules, and core genes were selected based on module connectivity. The top five core genes with the highest connectivity and their interaction relationships were visualized using Cytoscape software (Shannon et al., 2003) (v3.10.0) to clearly illustrate the hub nodes in the regulatory network.

### 2.6 Non-targeted metabolomics detection method

Plant tissue samples were accurately weighed at 25 mg and ground in liquid nitrogen. After adding 500 μL of extraction solution, the samples were vortexed and homogenized at 35 Hz for 4 minutes, followed by ice-water bath sonication for 5 minutes (this cycle was repeated 3 times). The samples were then frozen at -40 °C for 1 hour, centrifuged at 4 °C, and the supernatant was collected and filtered for analysis. For all samples with a sample size ≥ 12, 10 μL of supernatant was mixed to prepare a composite quality control sample. LC-MS/MS analysis was performed using a Thermo Vanquish UHPLC system coupled with an Orbitrap Exploris 120 mass spectrometer. The chromatographic column was a Phenomenex Kinetex C18 (2.1×100 mm, 2.6 μm), with the mobile phase consisting of 0.01% aqueous acetic acid solution (A) and isopropanol:acetonitrile (1:1, v/v) (B). The column temperature was set to 25 °C, and 2 μL was injected into the system. A gradient elution program was used (0-0.5 min: 1% B, 0.5-4.0 min: increased to 99% B, 4.0-4.5 min: maintain 99% B, 4.5-4.55 min: return to initial conditions). Mass spectrometry was conducted in IDA mode with positive and negative ion spray voltages set at 3.8 kV and -3.4 kV, respectively, and the scanning range was 70–1200 Da. In the data processing phase, raw data were first converted to MzXML format using ProteoWizard, followed by feature quality control (removal of features with high RSD) and missing value filtering (keeping features with ≤50% missing values) using XCMS. Missing values were half-quantified by replacing them with half of the minimum value, and the data were normalized using total peak area normalization (Zhou et al., 2012).

### 2.7 Non-targeted metabolomics analysis method

Based on the combined mass spectrometry data from both positive and negative ion modes, principal component analysis (PCA) was first performed on all samples using the R package *gmodels* (v2.18.1) (Warnes et al., 2018), aimed at unsupervised assessment of overall data quality, natural separation trends between sample groups, and within-group variability. To identify significantly different metabolites between groups, orthogonal partial least squares discriminant analysis (OPLS-DA) combined with univariate testing was employed: an OPLS-DA model was constructed using the R package *ropls* (Thevenot, 2016), and the model validity was verified by permutation testing (n=1000). Metabolites with a variable importance in projection (VIP) ≥ 1 in the OPLS-DA model and P-values< 0.05 after T-test correction were defined as significantly different metabolites. Finally, to analyze the biological functions of the differential metabolites, they were mapped to the KEGG database for pathway annotation, and KEGG pathway enrichment analysis was performed using hypergeometric testing. After FDR correction, pathways with a Q-value ≤ 0.05 were considered significantly enriched.

### 2.8 Quantitative real-time PCR validation

To validate the transcriptomic data, three randomly selected hub genes and three key genes involved in flavonoid biosynthesis were analyzed by qRT-PCR. Total RNA was extracted from plant samples using the RNAprep Pure Polysaccharide Polyphenol Plant Total RNA Extraction Kit (Tiangen, China). First-strand cDNA was synthesized from total RNA using a commercial reverse transcription kit (Tiangen). The cotton UBQ7 gene was used as an internal reference(M. Wang et al., 2013). Quantitative PCR was performed in triplicate on an Applied Biosystems 7500 Fast Real-Time PCR System using the BlasTaq™ 2X qPCR Mix (Abm, Canada), according to the manufacturer’s instructions. Relative gene expression levels were calculated using the 2−ΔΔCt method. Primer sequences are provided in **Supplementary Table S4**. A comparison between transcriptomic expression patterns and qRT-PCR validation results is shown in **Supplementary Figure S2**. All figures were generated using GraphPad Prism version 10 (Swift, 1997).

## 3 Results

### 3.1 Phenotypic changes and physiological indicators of red-leaf cotton under different drought stress treatments

**Figure 1:**
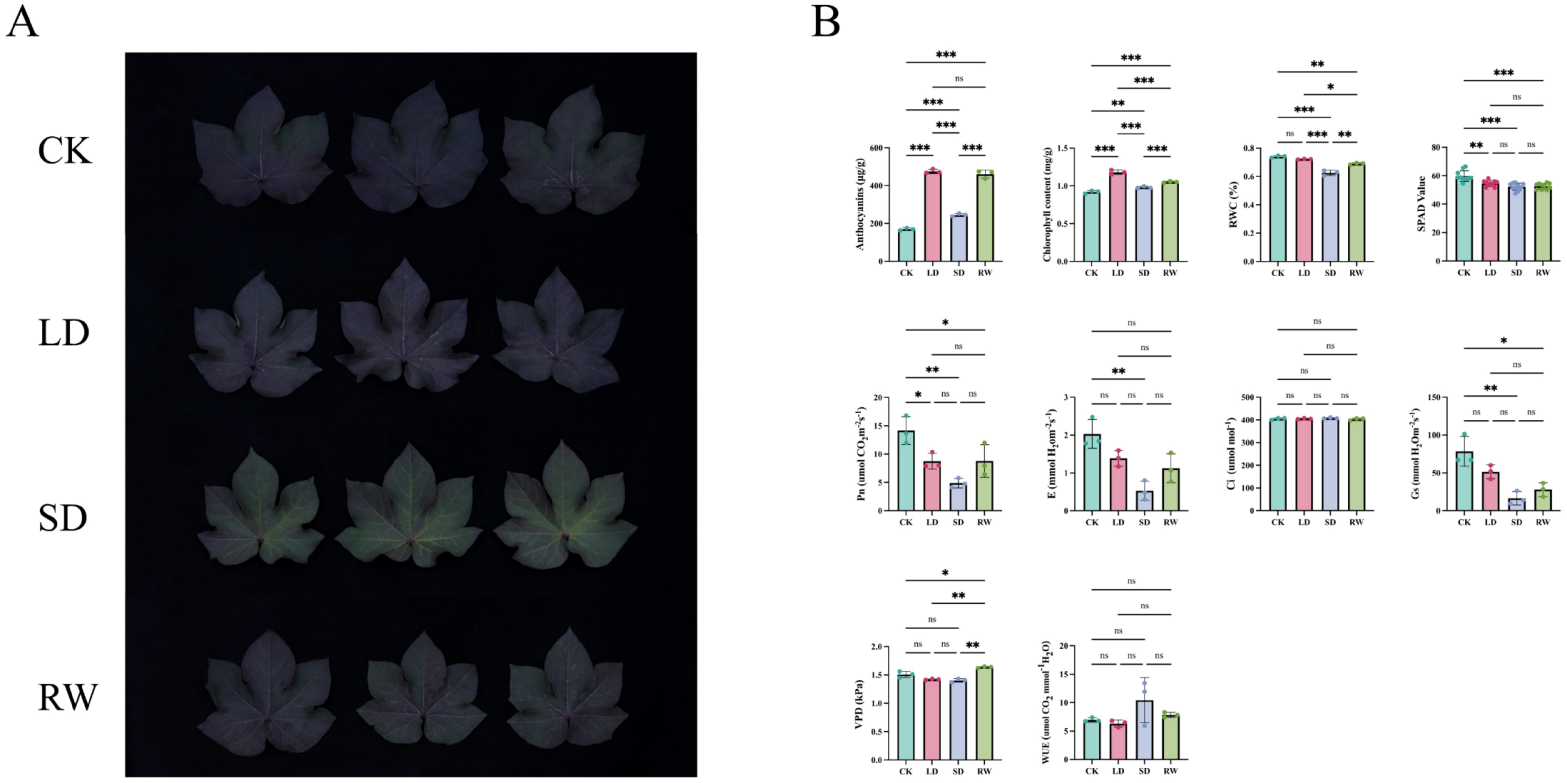
Phenotypic changes and physiological indicators of red-leaf cotton under different drought stress treatments. **(A)** Color changes of red-leaf cotton leaves under drought stress conditions. **(B)** Changes in physiological indicators under drought stress conditions. (Error bars represent the SE ± mean of three replicates, with *, **, and *** indicating P-values less than 0.05, 0.01, and 0.001, respectively).

We measured the phenotypic changes and physiological indicators of red-leaf cotton under different drought stress treatments. In the control group (CK), the leaves exhibited a light purple color, displaying the typical phenotype under normal water conditions. In the LD (mild drought) treatment group, the leaf color deepened to purple, indicating significant anthocyanin accumulation under mild drought stress. In the SD (severe drought) group, the leaf color lightened to a gray-green, indicating that severe drought stress significantly inhibited the plant’s photosynthesis. In the RW (rewatering) group, the leaves approached a purple-green color, suggesting that although rewatering was applied, the leaf color did not fully recover (**Figure 1A**).

Anthocyanin content in the LD group was significantly higher than in the other treatment groups, indicating that mild drought stress promotes anthocyanin accumulation and enhances the plant’s antioxidant capacity. In contrast, anthocyanin content in the SD group was significantly reduced, showing that anthocyanin degradation occurred under severe drought stress. The net photosynthetic rate (Pn) in the SD group was significantly lower than in the CK and LD groups, indicating that severe drought seriously inhibited the plant’s photosynthetic capacity. Meanwhile, relative water content (RWC) in the SD group was significantly reduced, showing a marked negative impact of drought stress on the leaf’s ability to retain water. After rewatering, these physiological indicators showed some recovery, but they did not return to CK levels, suggesting the long-term effects of drought on the plant.

Pn, transpiration rate (E), and stomatal conductance (Gs) were significantly lower in the SD group, indicating that drought inhibited the plant’s water use efficiency and gas exchange capacity. After rewatering, these indicators showed some recovery, but not full restoration, reflecting that plant physiological functions had not completely returned to normal levels after rehydration (**Figure 1B**). In summary, drought stress significantly affected multiple physiological indicators of red-leaf cotton, particularly in the SD group, where antioxidant capacity increased but photosynthesis and water retention were severely suppressed. Rewatering partially restored these physiological functions, but they did not fully recover, indicating that drought has a persistent and profound impact on the physiological functions of red-leaf cotton.

### 3.2 Transcriptome analysis of red-leaf cotton under different drought stress treatments

**Figure 2:**
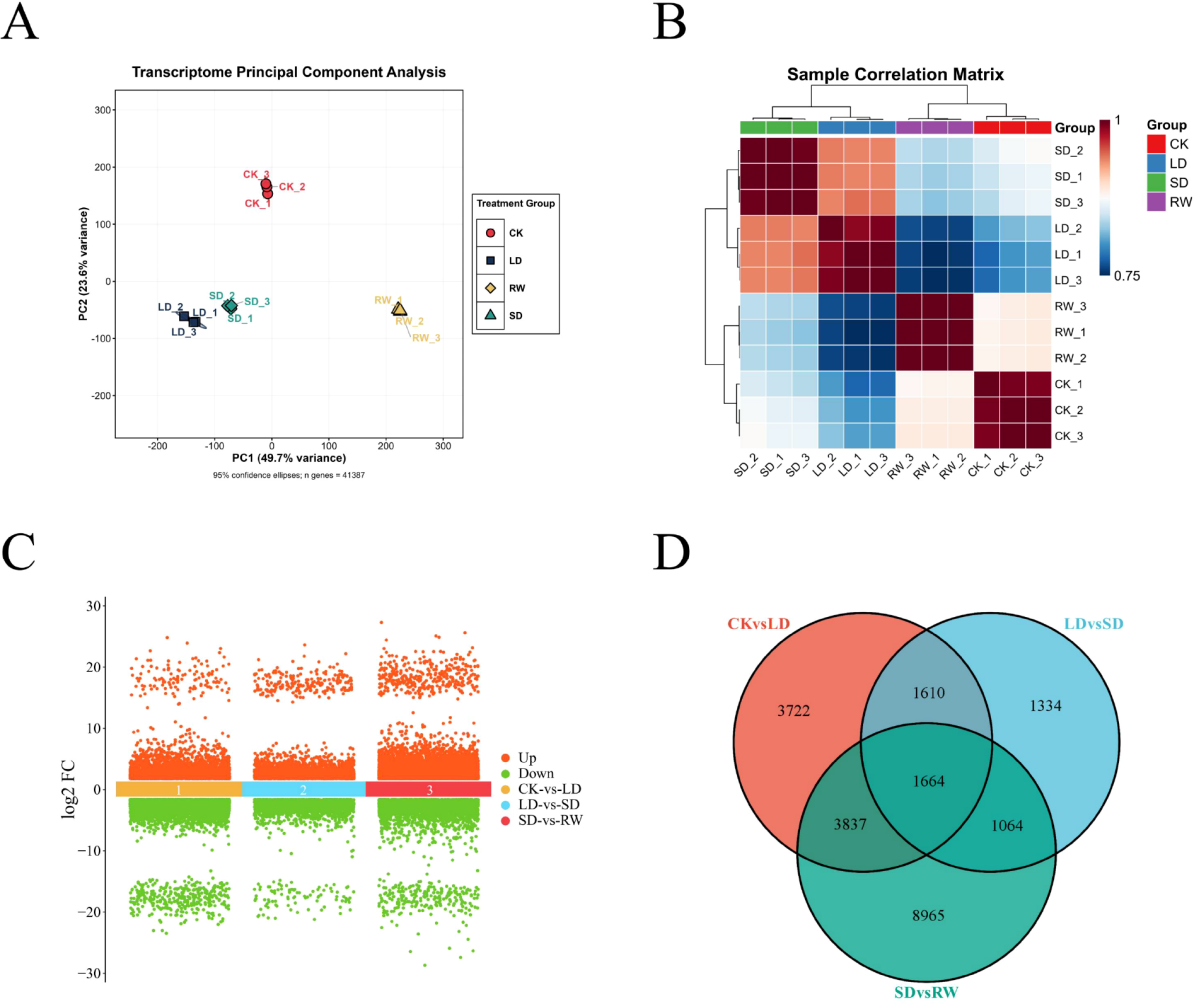
Transcriptome analysis of red-leaf cotton under different drought stress treatments. **(A)** Principal Component Analysis (PCA) results. **(B)** Sample correlation matrix. **(C)** Log2 fold changes of differentially expressed genes (DEGs) between different treatment groups. **(D)** Venn diagram showing the overlap of differentially expressed genes between different treatment groups.

Transcriptome analysis of red-leaf cotton under different drought stress treatments revealed significant differences between the groups. The PCA results showed that the samples from different treatment groups were distinctly distributed along principal component 1 (PC1) and principal component 2 (PC2), especially between the CK and other treatment groups (LD, SD, RW), indicating that drought stress and rewatering significantly affected transcriptome expression. Samples from the LD, SD, and RW groups were closer to each other, suggesting that mild and severe drought stresses had similar effects on the transcriptome, while the RW group was between the drought groups and the control group, showing distinct transcriptomic features (**Figure 2A**). Additionally, the sample correlation matrix showed a high degree of correlation within each group and significant differences between the groups, further confirming the impact of different treatment conditions on the transcriptome (**Figure 2B**).

Differential gene expression (DEG) analysis revealed that, in the comparison between the CK and LD groups, a total of 10,833 DEGs were identified, of which 4,246 genes were upregulated and 6,587 genes were downregulated. In the comparison between the LD and SD groups, 5,672 DEGs were identified, with 3,152 genes upregulated and 2,520 genes downregulated. In the comparison between the SD and RW groups, 15,530 DEGs were identified, with 11,791 genes upregulated and 3,739 genes downregulated (**Figure 2C**). Venn diagram analysis showed that 1,664 DEGs were common across the three groups, and these genes may be part of the core response mechanisms of the plant to different drought stress and rewatering treatments (**Figure 2D**).

In summary, drought stress (especially severe drought) had a significant impact on the transcriptome of red-leaf cotton. After rewatering, some gene expression was restored, but significant differences remained compared to the control group. This provides important genetic resources for further studies on the drought resistance mechanisms and recovery processes of red-leaf cotton.

### 3.3 KEGG and GO enrichment analysis of differentially expressed genes

**Figure 3:**
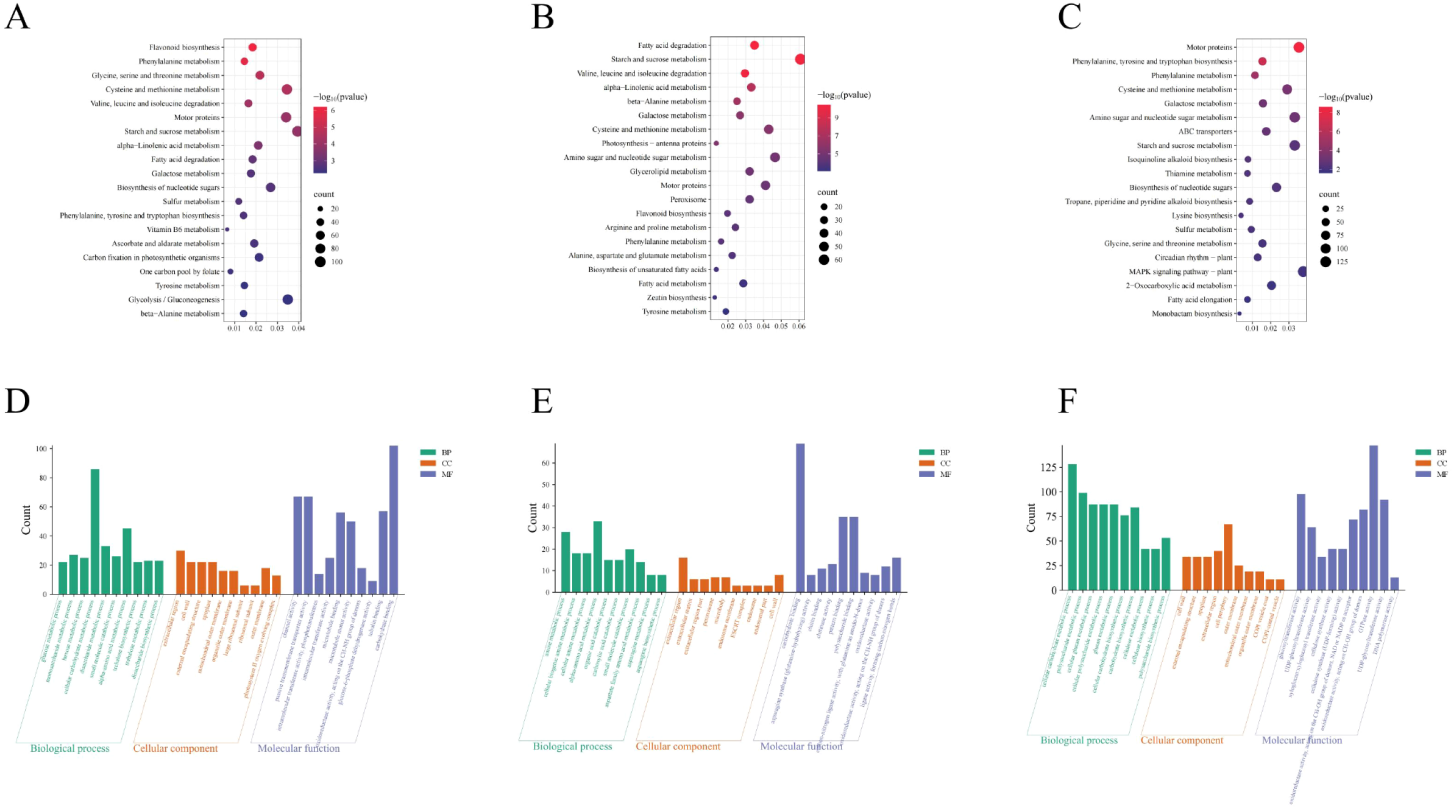
KEGG and GO enrichment analysis results of red-leaf cotton under different drought stress treatments. **(A)** KEGG pathway enrichment of differential genes between CK and LD. **(B)** KEGG pathway enrichment of differential genes between LD and SD. **(C)** KEGG pathway enrichment of differential genes between SD and RW. **(D)** GO enrichment results of differential genes between CK and LD. **(E)** GO enrichment results of differential genes between LD and SD. **(F)** GO enrichment results of differential genes between SD and RW.

KEGG and GO enrichment analyses of red-leaf cotton under different drought stress treatments revealed key metabolic pathways involved in drought response. In the comparison between CK and LD, the differentially expressed genes were enriched in pathways such as flavonoid biosynthesis, phenylalanine metabolism, fatty acid degradation, and amino acid metabolism, indicating that mild drought promoted the activation of antioxidant and osmoregulatory metabolic pathways. Although genes related to flavonoid biosynthesis were downregulated in the LD group, other drought-resistant pathways, such as fatty acid degradation, remained significantly enriched, which may help the plant cope with oxidative stress and water stress caused by mild drought. Additionally, the enrichment of amino acid metabolism and carbohydrate metabolism pathways reflects the plant’s ability to maintain cellular homeostasis by adjusting amino acid synthesis and carbohydrate reserves (**Figure 3A**).

In the comparison between LD and SD, differentially expressed genes were enriched in fatty acid degradation, starch and sucrose metabolism, and amino acid metabolism pathways. These pathways highlight how the plant enhances its drought resistance by regulating energy and water metabolism under severe drought stress. Particularly, the enrichment of fatty acid degradation and amino acid metabolism indicates that the plant strengthens cellular membrane and protein stability (**Figure 3B**). After rewatering (SD vs RW), the plant’s metabolic pathways showed some recovery, especially for key drought-resistant pathways like flavonoid biosynthesis, phenylalanine metabolism, and fatty acid metabolism. Moreover, the recovery of amino acid metabolism and carbohydrate metabolism pathways after rewatering suggests that the plant’s energy and osmoregulatory mechanisms improved following water restoration (**Figure 3C**).

GO enrichment analysis further revealed changes in biological processes, cellular components, and molecular functions under different treatments, especially enrichment in processes such as photosynthesis, metabolism, and antioxidant responses, indicating that drought stress significantly affected the photosynthesis, energy metabolism, and antioxidant capacity of red-leaf cotton (**Figures 3D-F**).

In summary, red-leaf cotton exerts its drought resistance by regulating multiple key metabolic pathways, such as flavonoid biosynthesis, phenylalanine metabolism, fatty acid degradation, and amino acid metabolism, under drought stress.

### 3.4 TF analysis

**Figure 4:**
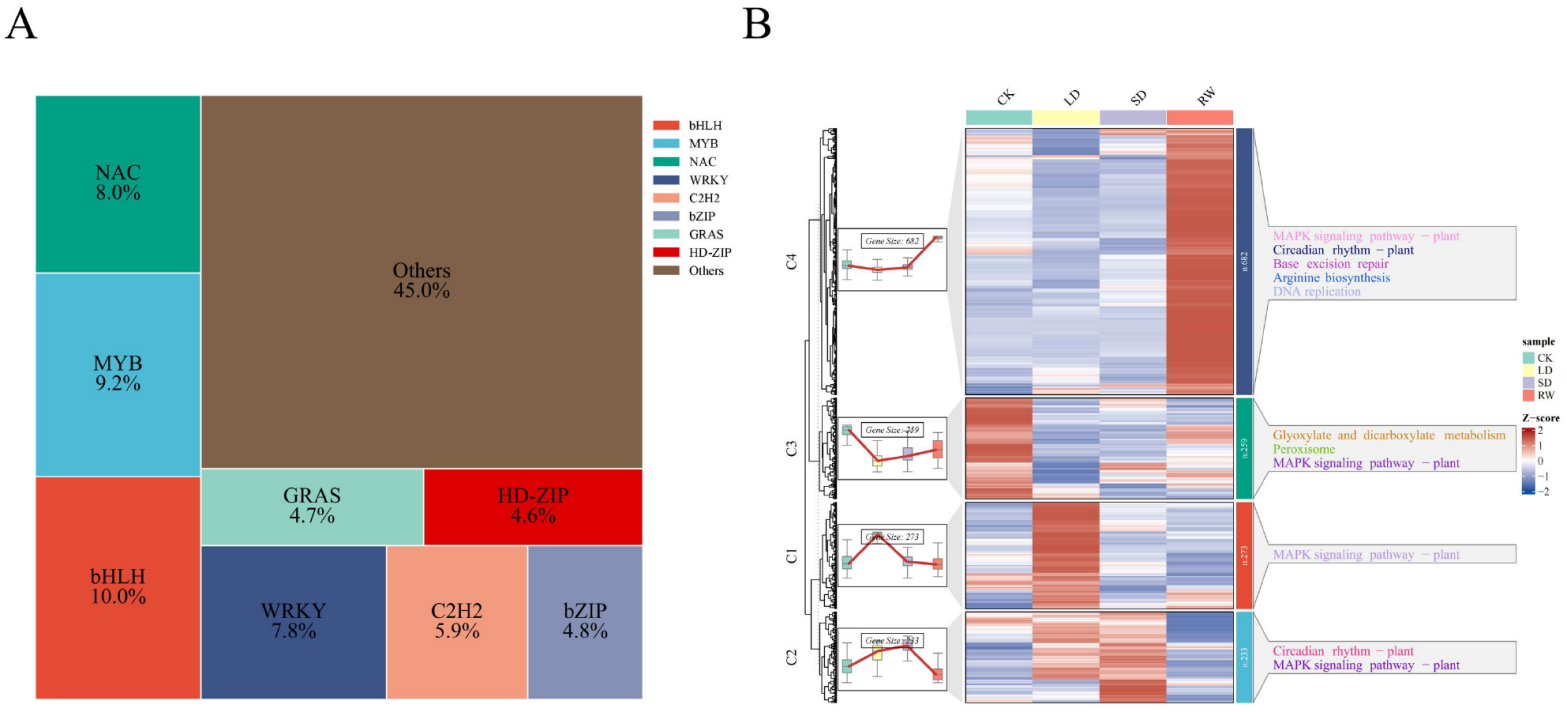
Clustering analysis and KEGG enrichment results of differentially expressed transcription factors (TFs) under different drought stress treatments in red-leaf cotton. **(A)** Proportion of differentially expressed transcription factor families. **(B)** Expression patterns and functional enrichment analysis of differentially expressed transcription factors.

In the differential expression analysis of transcription factors (TFs) in red-leaf cotton, a total of 1,448 differentially expressed TFs were identified, covering multiple key TF families, such as bHLH (10%), MYB (9.2%), and WRKY (7.8%) (**Figure 4A**). Cluster analysis grouped these differentially expressed TFs into four main clusters (C1 to C4), revealing their unique expression patterns under different treatments. The TFs in cluster C3 exhibited the highest expression in the CK group, with lower expression in the LD and SD groups, indicating that these TFs may play an important role under normal growth conditions and their expression is suppressed by drought stress. The TFs in cluster C1 showed the strongest expression under LD treatment, suggesting that these TFs may regulate the plant’s response to mild drought stress. TFs in cluster C2 had higher expression levels under both LD and SD treatments, with particularly strong upregulation in the SD group, indicating that these TFs are involved in the plant’s response to severe drought stress. The TFs in cluster C4 exhibited the highest expression in the RW group, suggesting that these TFs play a role in recovery after rewatering, helping the plant restore its physiological functions.

KEGG enrichment analysis revealed that these differentially expressed TFs were enriched in multiple important metabolic and signaling pathways, particularly the MAPK signaling pathway, circadian rhythm, DNA replication, and glycerol metabolism (**Figure 4B**). This suggests that transcription factors play a key role in the plant’s drought response, metabolic regulation, and recovery process, especially in regulating the plant’s metabolic function and drought resistance during recovery after rewatering.

### 3.5 WGCNA analysis

**Figure 5:**
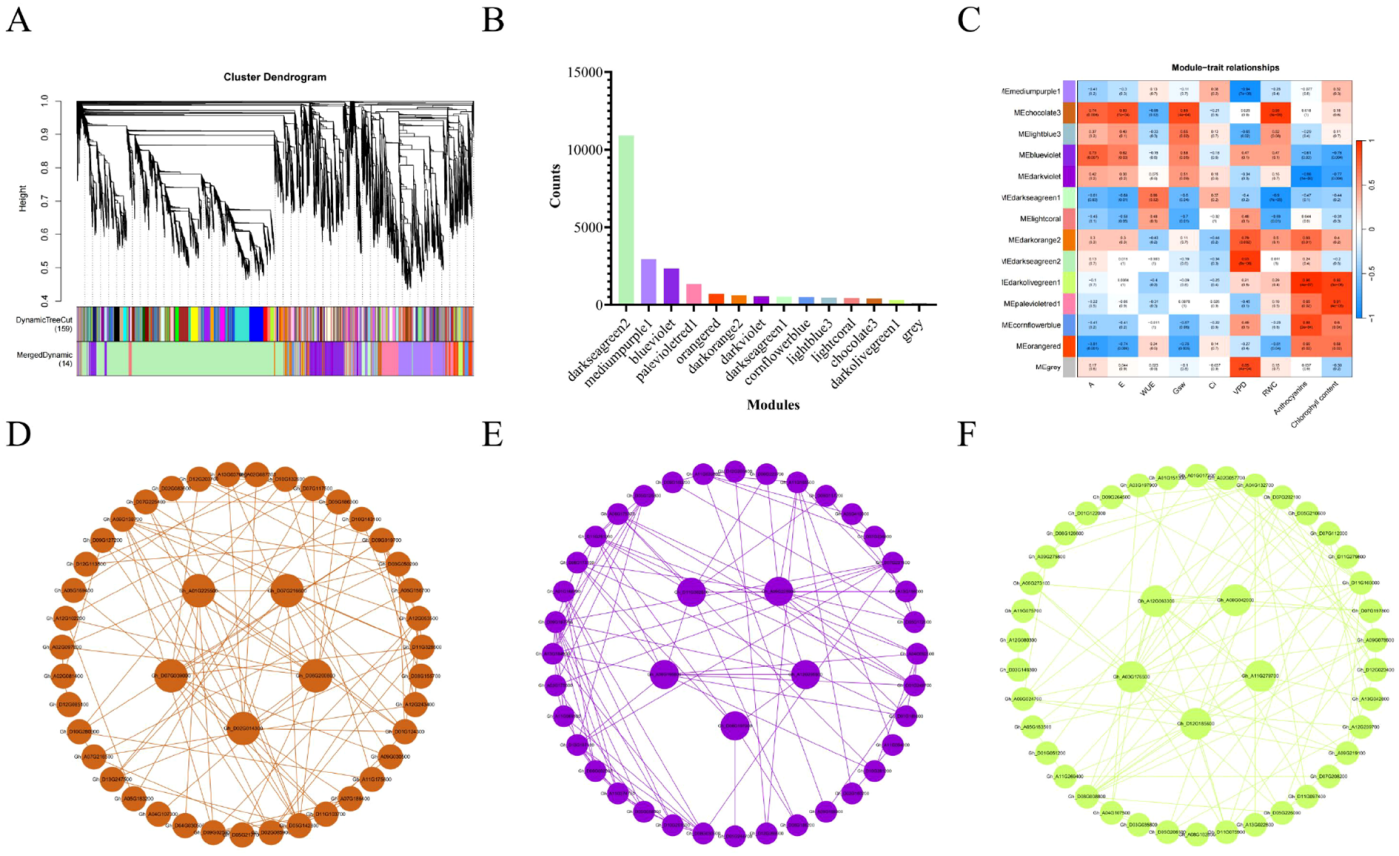
WGCNA analysis of red-leaf cotton and its correlation with physiological phenotypes. **(A)** WGCNA module hierarchical clustering dendrogram. **(B)** Distribution of gene numbers across modules. **(C)** Heatmap of correlations and significance between samples and modules. **(D-F)** Visualization of the three modules most strongly associated with RWC and anthocyanin content, and the top 5 genes with the strongest connectivity.

To study the gene regulatory networks involved in cotton’s response to drought stress, we constructed a co-expression network using weighted gene co-expression network analysis (WGCNA) with 22,195 differentially expressed genes (DEGs). These 22,195 DEGs were divided into 14 modules (**Figure 5A**), with the darkseagreen2 module containing the largest number of genes (10,923 genes) (**Figure 5B**). Correlation analysis with physiological indicators (such as relative water content (RWC) and anthocyanin content) identified the three modules most strongly correlated with these physiological traits. The chocolate3 module was positively correlated with RWC (0.95), the darkviolet module was negatively correlated with anthocyanin content (-0.96), and the darkolivegreen1 module was positively correlated with anthocyanin content (0.96) (**Figure 5C**), suggesting that these modules play important roles in the drought regulation and physiological responses of red-leaf cotton.

For these three modules most strongly associated with RWC and anthocyanin content, further visualization was performed using Cytoscape. The top 5 genes with the strongest connectivity in each module were selected as hub genes, and their gene interaction networks were displayed. In the chocolate3 module, hub genes included Gh_D07G039000, which encodes a glutamate-gated receptor potentially acting as a non-selective cation channel involved in plant ion channel regulation; Gh_A01G225500 and Gh_D07G216600, which encode LRR receptor-like serine/threonine protein kinases and potentially inactive leucine-rich repeat receptor-like protein kinases, playing important roles in plant immunity and environmental adaptation; Gh_D06G200800, which is associated with pathogen defense and leaf cell death regulation; and Gh_D02G014300, encoding a domain of unknown function (DUF4413), possibly involved in plant stress responses (**Figure 5D**).

In the darkviolet module, Gh_A09G225900 is involved in the interconversion of serine and glycine, helping the plant regulate amino acid balance. Several heat shock protein 70 family genes (e.g., Gh_D06G197900, Gh_D11G382600, Gh_A06G195800) indicate their critical roles in responding to drought and heat stress. Gh_A12G295500 encodes ethanolamine N-methyltransferase, possibly regulating drought-related physiological processes (**Figure 5E**).

In the darkolivegreen1 module, Gh_A03G176500 encodes an NAC transcription factor that regulates the expression of drought tolerance genes; Gh_A12G063300 is involved in the isomerization of citric acid to isocitric acid, regulating energy metabolism; Gh_D12G185600 is a protein kinase involved in signal transduction; Gh_A11G279700 is a light-inducible protein involved in light and drought responses; and Gh_A08G042000 encodes S-adenosylmethionine decarboxylase, regulating plant growth and metabolic responses (**Figure 5F**). These hub genes play key roles in the drought response, metabolic regulation, and recovery process of red-leaf cotton.

### 3.6 Metabolomics analysis of differential metabolites

**Figure 6:**
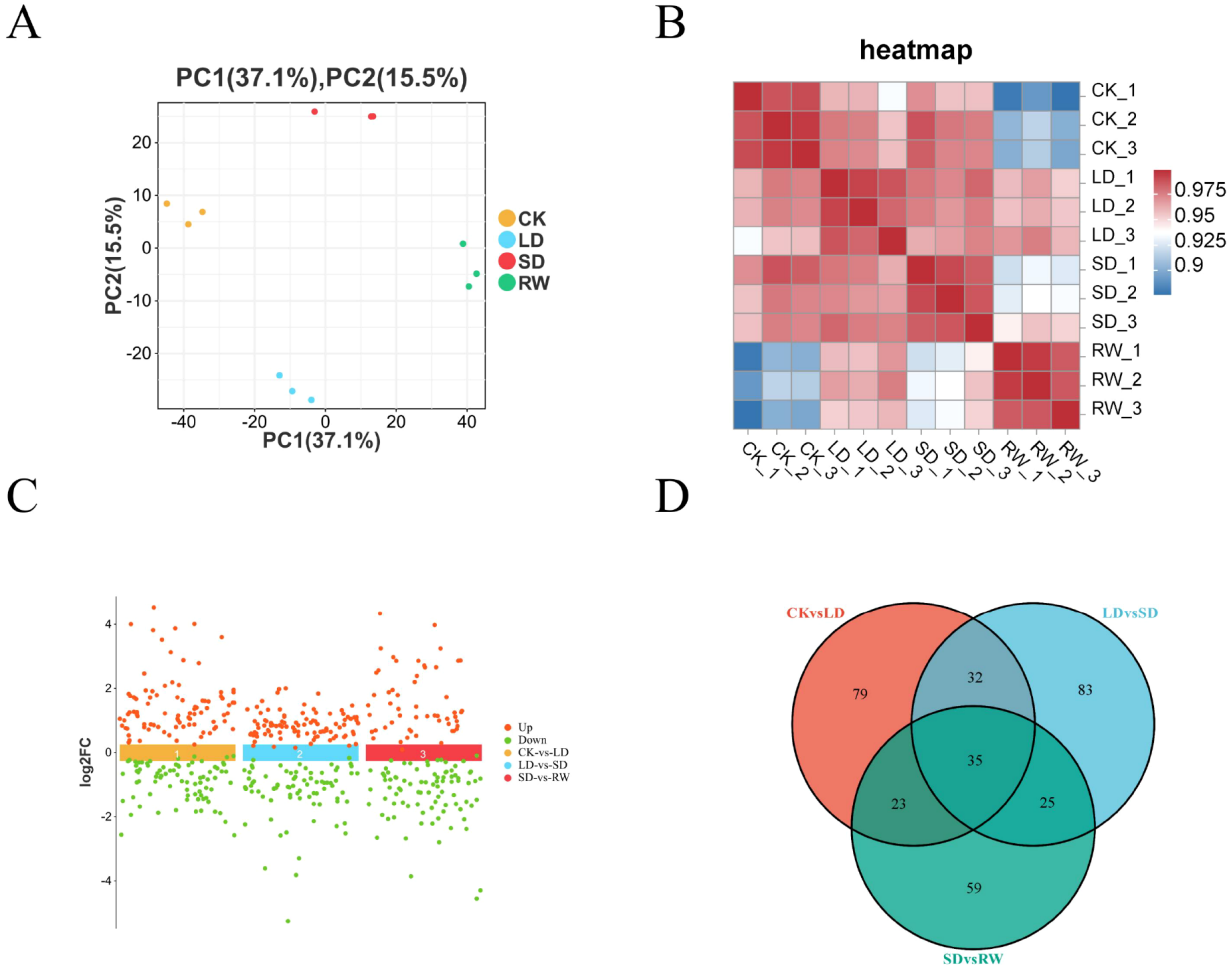
Sample correlation and differential metabolite analysis of red-leaf cotton. **(A)** Principal component analysis (PCA) showing metabolic differences between different treatment groups (CK, LD, SD, RW). **(B)** Metabolic sample correlation matrix. **(C)** Volcano plot showing significant differential metabolites between different treatment groups. **(D)** Venn diagram showing common and unique differential metabolites between different groups.

In the differential metabolite analysis of red-leaf cotton, principal component analysis (PCA) was first performed to visualize the metabolite data of different treatment groups (**Figure 6A**). The PCA results showed clear differences in the metabolite profiles between the treatment groups, particularly between the control group (CK) and the drought treatment groups (**Figure 6B**). The volcano plot (**Figure 6C**) further revealed significant changes in differential metabolites between the treatment groups, especially under severe drought stress, where many metabolites showed significant upregulation or downregulation. The Venn diagram (**Figure 6D**) displayed the common differential metabolites between the groups. In the comparison between CK and LD, 169 differential metabolites were identified; in the comparison between LD and SD, 175 differential metabolites were identified; and in the comparison between SD and RW, 142 differential metabolites were identified. The changes in these differential metabolites highlighted the significant impact of drought stress on the metabolism of red-leaf cotton.

### 3.7 KEGG enrichment analysis of differential metabolites

**Figure 7:**
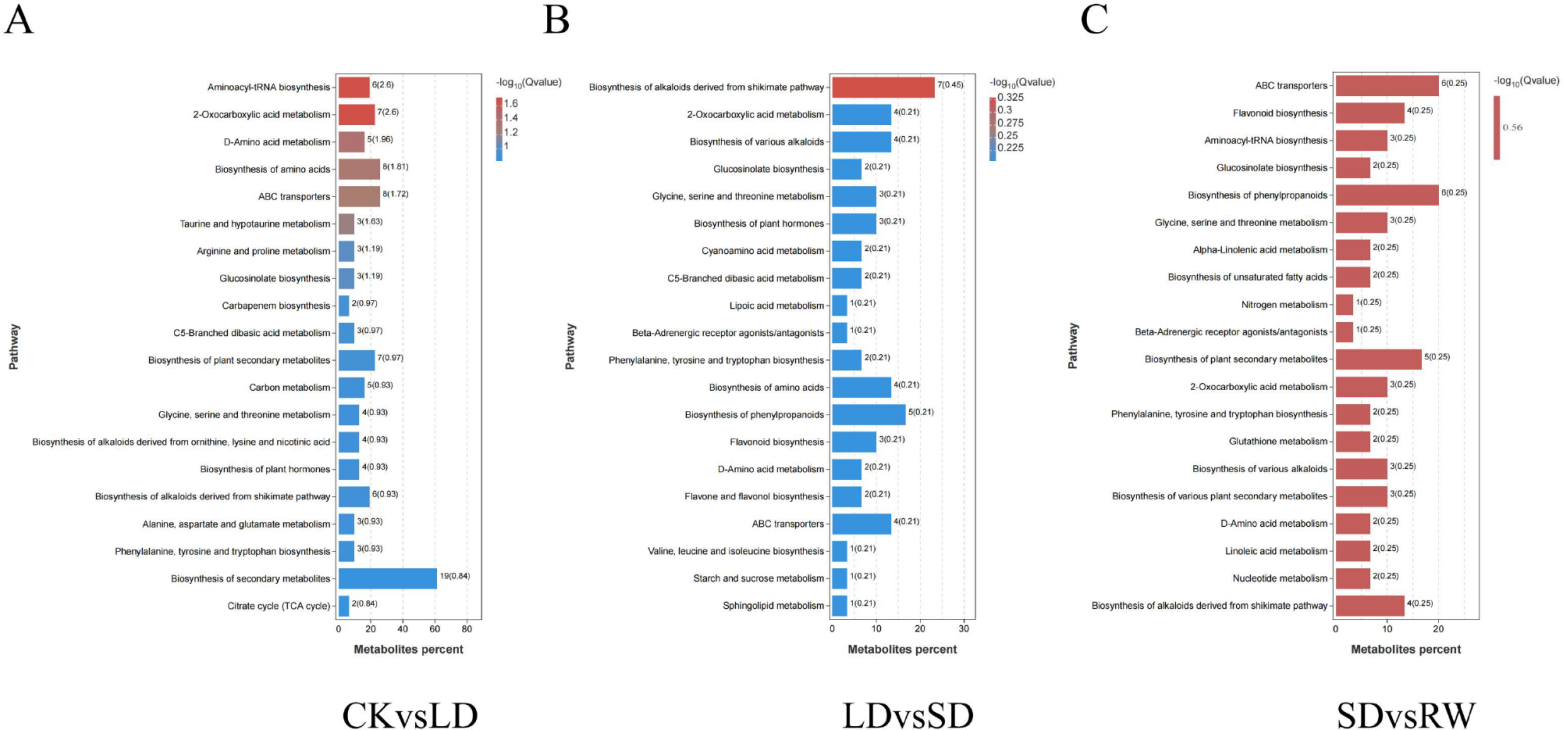
KEGG pathway enrichment analysis of differential metabolites in red-leaf cotton under different drought stress treatments. **(A)** KEGG pathways enriched in differential metabolites between CK and LD. **(B)** KEGG pathways enriched in differential metabolites between LD and SD. **(C)** KEGG pathways enriched in differential metabolites between SD and RW.

Non-targeted metabolomics analysis was performed to investigate the dynamic changes in metabolites during drought stress and rewatering recovery in red-leaf cotton, followed by KEGG pathway enrichment analysis. The results indicate that the drought resistance of red-leaf cotton relies on a highly dynamic and stage-specific metabolic network regulation. During the mild drought phase (CK vs. LD), the metabolic response was characterized by significant enrichment in amino acid biosynthesis (such as aminoacyl-tRNA biosynthesis, arginine and proline metabolism), plant hormone biosynthesis, and ABC transporter pathways (**Figure 7A**). This suggests that the plant establishes basic resistance by rapidly synthesizing osmoregulatory substances and initiating stress signaling pathways.

As drought intensified (LD vs. SD), the metabolic focus shifted towards multi-layered chemical defense systems. Significant enrichment was observed in flavonoid biosynthesis, alkaloid synthesis derived from the cinnamic acid pathway, and glucosinolate biosynthesis pathways (**Figure 7B**), revealing the plant’s strategy of co-activating multiple secondary metabolic pathways to cope with oxidative and biological stress under severe stress conditions. However, it is noteworthy that although the flavonoid biosynthesis pathway was enriched, the accumulation of its key metabolites (such as quercetin and kaempferol glycosides) was significantly downregulated. This reflects that under extreme resource limitation, the rapid consumption of flavonoids (for reactive oxygen species scavenging) outweighed its synthesis capacity, or carbon skeleton resources were preferentially allocated to other more direct survival pathways (such as cell wall reinforcement), highlighting the metabolic trade-offs that plants undergo under extreme stress.

After rewatering (SD vs. RW), the metabolic profile underwent a fundamental shift, showing strong features of repair and reconstruction. The flavonoid biosynthesis pathway was strongly reactivated and became one of the most significantly enriched pathways, with its levels significantly increasing compared to the drought period, accompanied by the upregulation of glutathione metabolism and unsaturated fatty acid biosynthesis (**Figure 7C**). This indicates that after the alleviation of water stress, the plant prioritized resources to its antioxidant repair systems (flavonoids and glutathione synergistically) and membrane system stability restoration. The substantial synthesis of flavonoids at this stage is aimed at clearing oxidative damage accumulated during drought and preparing “metabolic reserves” for potential subsequent stresses.

### 3.8 Analysis of significantly differential metabolites

**Figure 8:**
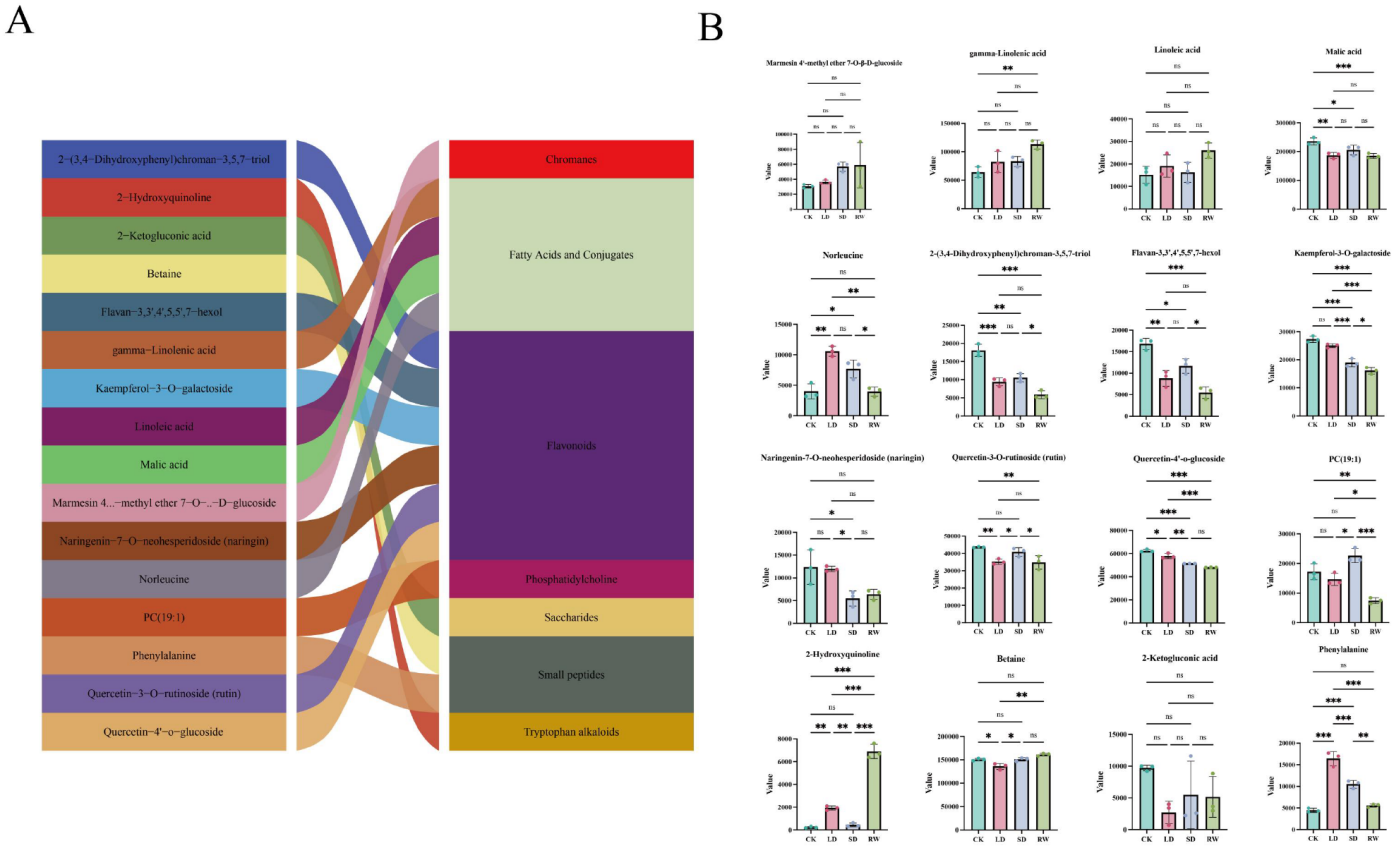
Analysis of 16 differential metabolites with VIP values greater than 5 and p-values less than 0.05 in different treatment groups of red-leaf cotton. **(A)** Sankey diagram showing the classification of metabolites across different treatment groups. **(B)** Expression levels of metabolites in different treatment groups (CK, LD, SD, RW). (Error bars represent SE ± mean of three replicates, with *, **, *** indicating P-values less than 0.05, 0.01, and 0.001, respectively).

Based on the criteria of Variable Importance in Projection (VIP > 5) and significance testing (p< 0.05), we identified 16 highly significant differential metabolites from the metabolomic data of red-leaf cotton. These metabolites were classified into 7 groups based on their categories, with 6 metabolites belonging to flavonoids, and the others being fatty acids, amino acids, phenolic compounds, and small molecule metabolites (**Figure 8A**).

In the LD group, the phenylalanine content was significantly elevated by 268% compared to CK, along with a sharp increase in 2-hydroxyquinoline accumulation (up 774%), which together reveal the rapid activation of early secondary metabolic pathways. This likely serves as the metabolic basis for enhanced flavonoid and anthocyanin synthesis, manifesting as the typical red-leaf phenotype. As drought intensified to severe levels, red-leaf cotton exhibited an efficient membrane protection mechanism: the membrane lipid phosphatidylcholine PC(19:1) showed a significant increase compared to the LD stage, while polyunsaturated fatty acid γ-linolenic acid continuously accumulated, helping maintain membrane stability. Notably, the osmotic regulator betaine reached its highest level after rewatering, demonstrating its strong osmoregulatory and damage repair capacity. After rewatering, the explosive accumulation of 2-hydroxyquinoline (increased by 1475% compared to SD) suggested that red-leaf cotton may possess a unique “stress memory” or early warning defense mechanism. However, some glycosylated flavonoids (such as quercetin-4’-O-glucoside) decreased in content under prolonged stress, indicating that they may be prioritized for consumption as an antioxidant “metabolic pool” to mitigate oxidative damage (**Figure 8B**).

In summary, the drought resistance of red-leaf cotton not only stems from its physical and antioxidant defenses enhanced by phenylpropanoid metabolism in the early stages (reflected in the red-leaf phenotype), but also relies on precise membrane lipid remodeling, osmoregulation, and the active repair and early warning systems after rewatering. These coordinated metabolic pathways together form a multi-layered, dynamic adaptive network that enables red-leaf cotton to cope with water stress.

### 3.9 Flavonoid pathway analysis

**Figure 9:**
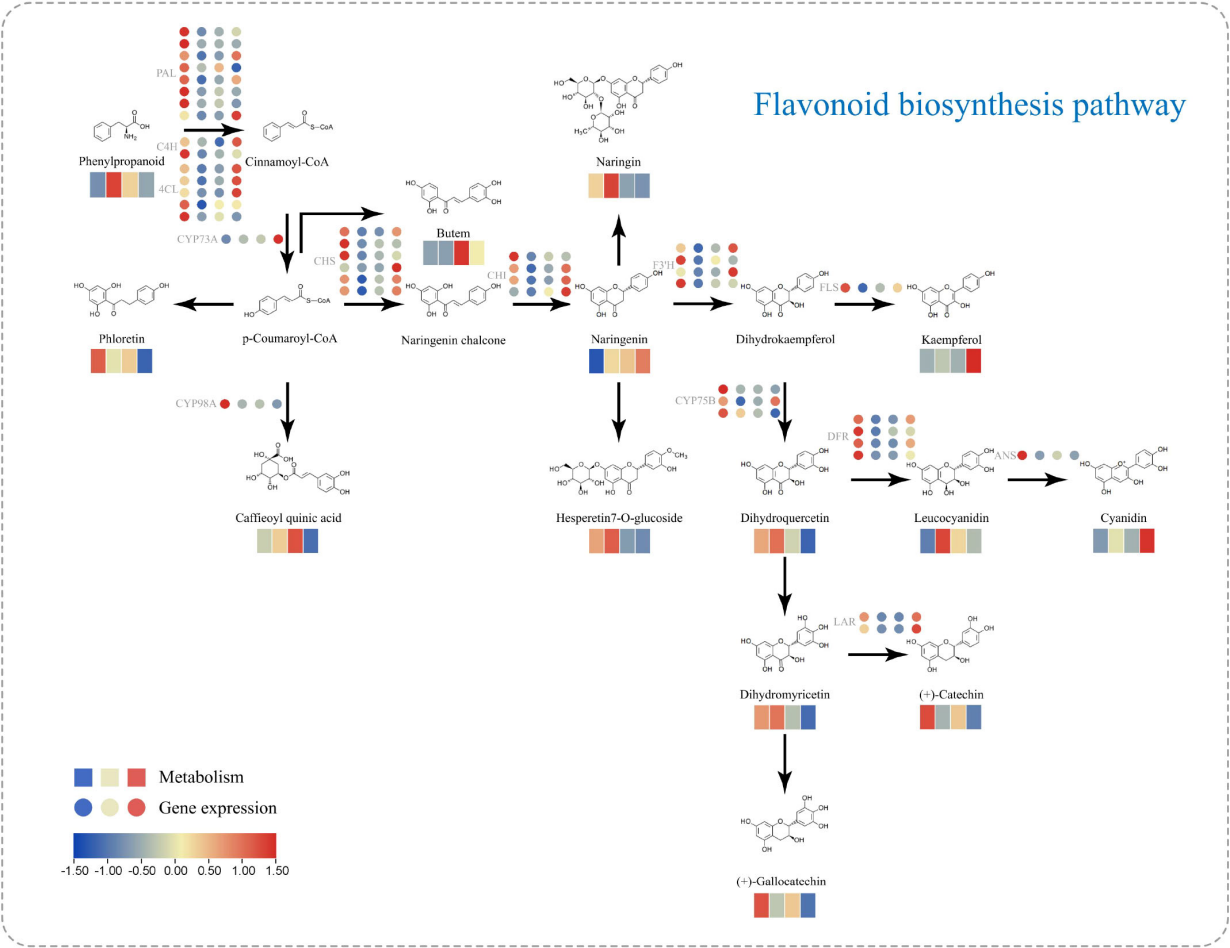
Key genes and differential metabolite analysis of the flavonoid biosynthesis pathway in red-leaf cotton under drought stress.

Under drought stress, the flavonoid biosynthesis pathway in red-leaf cotton showed significant inhibition and exhibited changes in metabolites related to antioxidant capacity. In the flavonoid biosynthesis pathway, key genes such as PAL (phenylalanine ammonia-lyase), C4H (cinnamate-4-hydroxylase), CHS (chalcone synthase), F3H (flavanone-3-hydroxylase), and CYP450 genes (e.g., CYP73A, CYP98A, CYP75B1) play crucial roles. Under drought stress, the expression of these key genes was significantly suppressed, especially in the SD group, where the expression levels of these genes were generally reduced. PAL, being the rate-limiting enzyme in flavonoid biosynthesis, showed a significant decline in expression under SD treatment, directly affecting the conversion of phenylalanine to cinnamic acid, thereby inhibiting the initial step of flavonoid synthesis. The expression of C4H and CHS genes also significantly decreased, particularly in the SD group, resulting in reduced synthesis of precursors like cinnamic acid and p-coumaric acid, further affecting the synthesis of flavonoid compounds. Additionally, CYP450 genes showed significant downregulation under drought stress, especially CYP75B1 and CYP73A. These P450 enzymes play an oxidative role in flavonoid biosynthesis, contributing to the modification and diversification of flavonoids. The reduced expression of these genes under drought conditions indicates that red-leaf cotton suppresses the diversity and synthesis of flavonoids when coping with drought stress (**Figure 9**).

Under drought stress, the synthesis of flavonoid metabolites in red-leaf cotton also significantly decreased. Particularly under severe drought (SD) conditions, the synthesis of flavonoids such as Naringin (naringin) and Kaempferol (kaempferol) decreased significantly, reflecting a decline in the plant’s antioxidant capacity. The synthesis of Naringin significantly decreased in the SD group, which was consistent with the downregulation of genes like C4H and CHS. Naringin is an important glycosylated flavonoid, and its reduced synthesis may indicate that red-leaf cotton prioritized resources for water maintenance and growth during drought, while reducing the synthesis of antioxidant substances. Kaempferol, another important antioxidant flavonoid, also showed significant reduction in synthesis under drought conditions. The reduction of Kaempferol may lead to increased oxidative stress during photosynthesis and drought stress in red-leaf cotton. After rewatering, red-leaf cotton initiated the recovery of the flavonoid biosynthesis pathway by restoring water. As water was restored, the expression of genes like PAL, C4H, CHS, and F3H gradually increased, leading to the restoration of flavonoid synthesis. After rewatering, red-leaf cotton not only restored the synthesis of anthocyanins but also the synthesis of other flavonoid metabolites such as Naringin and Kaempferol. Naringin gradually increased after rewatering, recovering from the low levels in the SD group to levels close to the control group, indicating that red-leaf cotton regained its ability to cope with oxidative stress after rewatering. The recovery of Kaempferol was particularly notable, as its content returned to levels close to the control group, further enhancing the antioxidant capacity of red-leaf cotton. This suggests that by restoring flavonoid biosynthesis, red-leaf cotton improved its antioxidant capacity and helped mitigate oxidative damage during the recovery process after rewatering.

## 4 Discussion

### 4.1 Core regulatory mechanism: systemic shutdown of the flavonoid pathway under drought stress

The systematic reprogramming of the flavonoid/anthocyanin biosynthesis pathway is the direct molecular engine behind the dynamic phenotypic change of red-leaf cotton from “red to green to red” under drought stress. Our multi-omics data clearly reveal the stage-specific changes in this pathway at the transcriptional, metabolic, and regulatory levels. Starting from mild drought, key genes in this pathway were coordinately downregulated: not only were upstream phenylpropanoid genes (PAL, 4CL) significantly downregulated, but flavonoid backbone biosynthesis genes (CHS, CHI, F3H) and downstream modification genes (such as CYP450 family members) were also simultaneously suppressed(W. Liu et al., 2021). This “full pathway suppression” regulatory pattern suggests that the plant may systematically shut down this high-energy-consuming secondary metabolic pathway through a centralized transcriptional regulatory network (Soubeyrand et al., 2018). Notably, this systemic transcriptional suppression was significantly reversed early after rewatering. Seven days after rewatering, although the leaf phenotype had not fully restored to red, the metabolic pathways were rapidly reactivated at the transcriptional level. The expression of upstream phenylpropanoid genes such as PAL and 4CL increased first, followed by a significant upregulation of core biosynthesis genes such as CHS, CHI, F3H, and CYP450 family modification genes. This hierarchical transcriptional recovery laid the molecular foundation for the progressive accumulation of anthocyanins, ultimately driving the complete reversal of the leaf phenotype from “green” to “red” after 20 days of rewatering (**Supplementary Figure S1**).

At the metabolite level, the response showed more refined temporal characteristics. The suppression of gene expression directly led to the blockade of metabolic flux and changes in the metabolic pool (Anzano et al., 2022). Intermediate metabolites in the pathway, such as the derivative of hesperetin chalcone, hesperidin, and downstream flavonol glycosides like kaempferol, showed significant decreases at the SD stage. These compounds are not only precursors or branch products of anthocyanin synthesis but are also important antioxidants (Guven et al., 2019). Their reduction, along with the shutdown of the anthocyanin synthesis endpoint, collectively contributed to the decrease in leaf red pigment and the “greening” phenomenon.

In contrast, purple-leaved kale-type rapeseed maintains its purple color under drought conditions by upregulating the expression of key flavonoid synthesis genes (CHS, DFR, ANS, etc.) to increase anthocyanin synthesis (Chen et al., 2025). However, under severe drought, the expression of key genes in the flavonoid synthesis pathway is also inhibited. The unique aspect of red-leaved cotton lies in its dramatic phenotypic response: due to its anthocyanins being primarily induced and having low basal levels, the leaves exhibit a striking color change from red to green. In comparison, purple-leaved kale-type rapeseed, due to its extremely high constitutive anthocyanin content, maintains its purple color even when synthesis is inhibited, resulting in minimal phenotypic changes.

### 4.2 Phenotypic and physiological implications: “greening” as a comprehensive indicator of impaired cellular function

However, “greening” does not imply optimization of photosynthetic apparatus. In fact, the synchronous decrease in chlorophyll content and photosynthetic rate under severe drought suggests that this “green” is more likely a “pale green”. Under severe drought, disruptions in membrane lipid metabolism and the depletion of antioxidant systems lead to damage to the cell membrane integrity and loss of selective permeability (Su et al., 2025). This prevents the cells from maintaining turgor pressure, causing irreversible water loss. At this point, although the leaf appears “green” due to the loss of anthocyanins, its cells are already in a state of physiological dehydration, and the chloroplast structure may also be damaged (Chng et al., 2022). Anthocyanins are primarily stored in the vacuole, and their stable accumulation depends on the acidic pH environment of the vacuole and potential “vacuolar compartmentalization” (Cerqueira et al., 2023). Severe drought may reduce vacuolar membrane stability, alter vacuolar pH, or damage compartmentalized structures, accelerating the degradation or leakage of anthocyanins. As a result, even if there is residual synthetic potential, the red phenotype cannot be effectively maintained. The “passive exposure” of chlorophyll and the inactivation of photosynthetic functions: the fading of anthocyanins merely exposes the underlying chlorophyll color, but this is not a signal of enhanced photosynthetic function. On the contrary, accompanied by membrane system damage and oxidative stress, the chlorophyll content may actually decrease due to increased degradation, and the function of photosynthetic organs (such as photosystem II) is severely compromised, leading to a sharp decline in photosynthetic rate (Qiao et al., 2024). Therefore, “pale green” is essentially a pathological phenotype resulting from the loss of protective pigments (anthocyanins), degradation of photosynthetic pigments, and collapse of cell structures.

Flavonoid/anthocyanin synthesis is a highly energy-consuming secondary metabolic pathway that requires substantial amounts of phenylalanine, malonyl-CoA, ATP, and reducing power (NADPH)(J. Wu et al., 2023). Under adequate water, investment in this pathway can yield strong photoprotection and resistance to biotic stress. However, under severe drought, when carbon assimilation is severely hindered (Pn decreases by about 50%), and a water and energy crisis arises, continuing to maintain this high-energy pathway becomes a survival burden. Therefore, actively downregulating this pathway is likely a “life-saving” trade-off made by the plant at a systemic level: it releases the extremely limited carbon skeletons, nitrogen sources, and reducing power from pigment synthesis and redirects them to urgent needs to maintain basic cellular survival, including synthesizing osmotic regulators like betaine and proline to maintain turgor, increasing the proportion of unsaturated fatty acids to maintain membrane fluidity (Baccouch et al., 2023), and ensuring basic respiratory metabolism to produce the ATP required for life activities.

### 4.3 Restoration cascade process: structural repair precedes pigment accumulation in the rehydration response sequence

The rewatering process reversed this regulatory logic. As water supply was restored and survival pressure was alleviated, growth and long-term competitive demands became dominant again. The transcriptomic and metabolomic data from 7 days after rewatering showed that the flavonoid pathway had fully resumed at both the molecular and metabolic levels. At the metabolite level, early metabolic responses preceded delayed phenotypic changes. Among them, flavonoid pathway metabolites increased significantly 7 days after rehydration, while leaf color did not fully recover to red until 20 days after rehydration. This phenomenon, where metabolite accumulation precedes visible phenotypic changes, indicates that the metabolic flux of the flavonoid pathway was rapidly re-established after transcriptional recovery. The re-accumulation of metabolites was a necessary prerequisite for phenotypic recovery. However, the visible phenotype lagged significantly behind metabolite accumulation, because drought stress not only suppressed anthocyanin biosynthesis but also caused systemic damage to the storage sites for anthocyanins. Specifically, anthocyanins are mainly stored in the vacuole, and their stable accumulation depends on the integrity of the vacuolar membrane system and the suitable acidic pH environment. Severe drought-induced membrane lipid peroxidation and osmotic stress severely damaged vacuolar membrane integrity and proton pump function (Jafari et al., 2022). Even if the synthetic pathway is reactivated, newly synthesized anthocyanins are difficult to store effectively and promptly in vacuoles. Cell membrane damage needs to be repaired preferentially. Before the membrane system is fully restored, plants must devote their limited resources to rebuilding membrane lipids, such as the synthesis of unsaturated fatty acids and the accumulation of osmotic regulators, and this work must be completed before supporting the synthesis and storage of high-energy pigments. The restoration of organelle function, re-establishment of pH homeostasis, and the recovery of cellular structures all take time, which together constrain the efficient accumulation and coloration of anthocyanins. The change in leaf color from green to red during the rehydration phase marks a critical shift in the plant’s physiological state: from a survival state focused on maintaining vital activity under drought stress to a state after water recovery that prioritizes tissue repair and nutritional growth. Notably, the accumulation dynamics of certain metabolites (such as 2-hydroxyquinoline) after rehydration differ significantly from their response patterns during drought stress. This phenomenon suggests that prior drought stress may have permanently altered the regulatory baseline of related metabolic pathways, thereby placing the plant in a preparatory state with a continuously elevated basal defense level.

**Figure 10:**
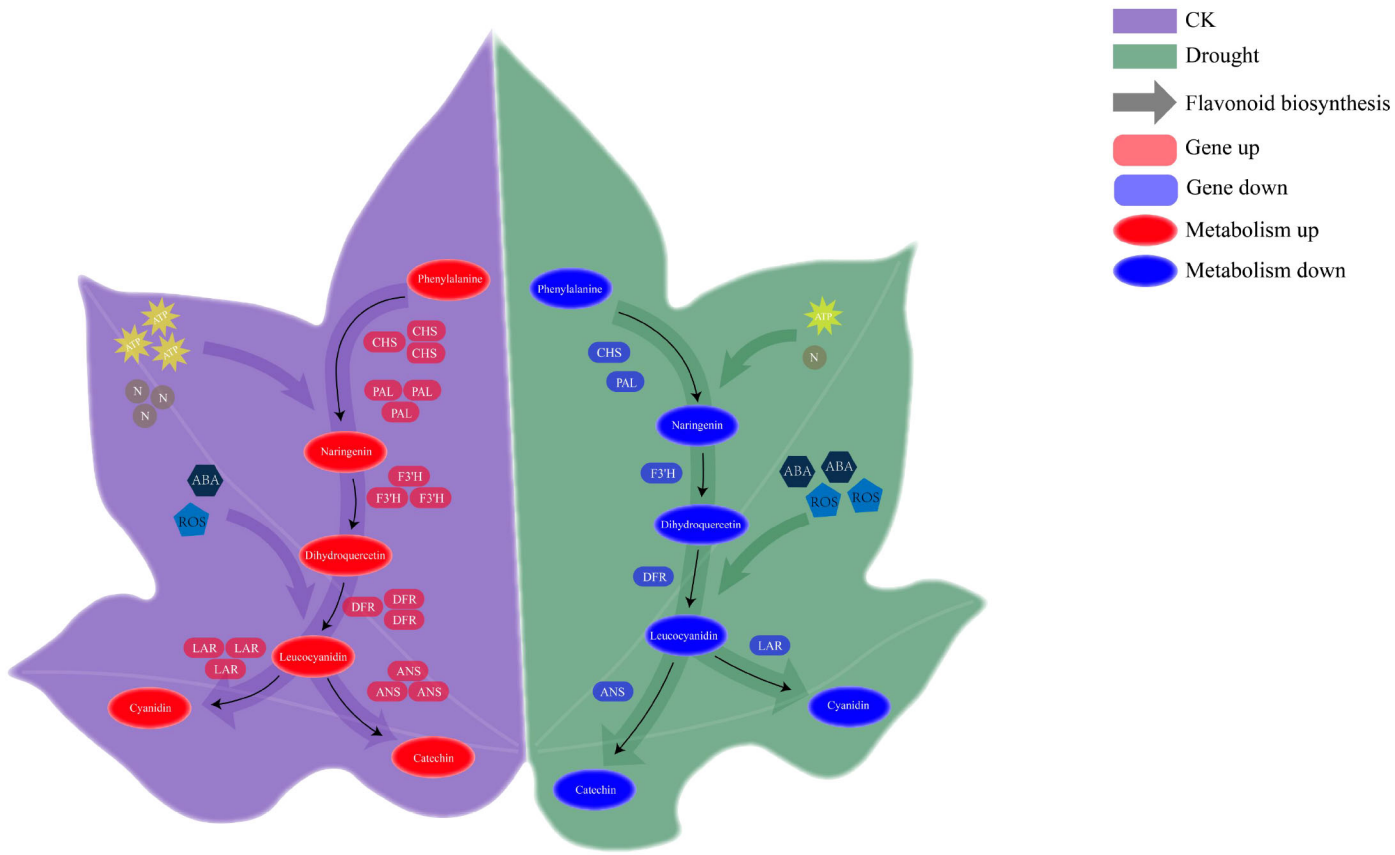
The mechanism by which red-leaf cotton regulates the flavonoid biosynthesis pathway to alter leaf color and participate in cotton drought stress regulation.

In conclusion, the leaf color change in red-leaf cotton is essentially the result of a reversible, networked “metabolic switch” that precisely regulates the flavonoid biosynthesis pathway (**Figure 10**). This switch is regulated by the intensity of drought stress, with its core objective being to make dynamic optimal decisions between “investing in defense (red)” and “ensuring survival (green).” The process from rapid metabolite accumulation 7 days after rewatering to full phenotypic recovery after 20 days not only reflects the rapid response capability of metabolic pathways but also reveals a complete recovery cascade from molecular reconstruction to cellular structural repair to phenotypic manifestation. This phased, priority-based recovery strategy—first rebuilding the metabolic foundation, then repairing cellular structures, and finally achieving phenotypic recovery—visually represents the plant’s sophisticated evolutionary adaptation to fluctuating drought environments.

## 5 Conclusions

This study reveals the unique dynamic leaf color changes (red-green-red) exhibited by red-leaf cotton during drought stress and rewatering, and through the combined analysis of transcriptomics and metabolomics, it delves into the molecular and biochemical mechanisms behind these changes. Under mild drought stress, red-leaf cotton enhances its antioxidant capacity by increasing anthocyanin accumulation, exhibiting strong photoprotection. However, under severe drought stress, the leaves turn green, with significant reductions in water retention and photosynthetic capacity, reflecting a shift in the plant’s survival strategy under extreme drought conditions. After rewatering, red-leaf cotton gradually restores anthocyanin synthesis by reactivating the flavonoid biosynthesis pathway, showing significant phenotypic recovery. However, this process involves a time lag, indicating that the reconstruction of the metabolic foundation after rewatering requires time. Transcriptomic analysis revealed significant gene expression reprogramming in red-leaf cotton under drought stress, particularly in genes related to key drought-resistant pathways such as flavonoid biosynthesis, phenylalanine metabolism, and fatty acid degradation. WGCNA analysis further identified gene modules closely associated with anthocyanin synthesis and water retention capacity, which play crucial regulatory roles during both drought stress and rewatering. Metabolomic analysis showed that red-leaf cotton exhibited stage-specific, dynamic metabolic regulation at different drought stages, with the accumulation of metabolites like phenylalanine and 2-hydroxyquinoline in the mild drought stage providing essential precursors for anthocyanin synthesis. In the severe drought stage, the plant maintained cellular stability by adjusting membrane lipids and osmotic regulators, while flavonoid synthesis was suppressed. After rewatering, the recovery of metabolites provided the basis for phenotypic recovery, indicating that red-leaf cotton possesses strong metabolic repair and stress memory capabilities.

In conclusion, red-leaf cotton dynamically adjusts the “switch” of flavonoid biosynthesis through precise metabolic regulation to cope with the physiological challenges of drought stress and water recovery. This mechanism not only reveals the plant’s adaptive strategy to water stress but also provides important theoretical support for the breeding of drought-resistant cotton varieties. Future research should further explore the functions of these key metabolites and genes and how they work together to optimize the plant’s drought resistance and recovery ability, offering new insights for crop resilience improvement.

## Data Availability

The original contributions presented in the study are publicly available. This data can be found here: NCBI BioProject, accession PRJNA1380067.
