# Supplementary figures and images for: Transcriptomics and non-targeted metabolomics reveal the mechanisms of leaf color changes in red-leaf cotton under drought stress and rewatering
Source: Front Plant Sci. 2026 Feb 16;17:1766818. doi: 10.3389/fpls.2026.1766818 (PMC12951637; doi:10.3389/fpls.2026.1766818)

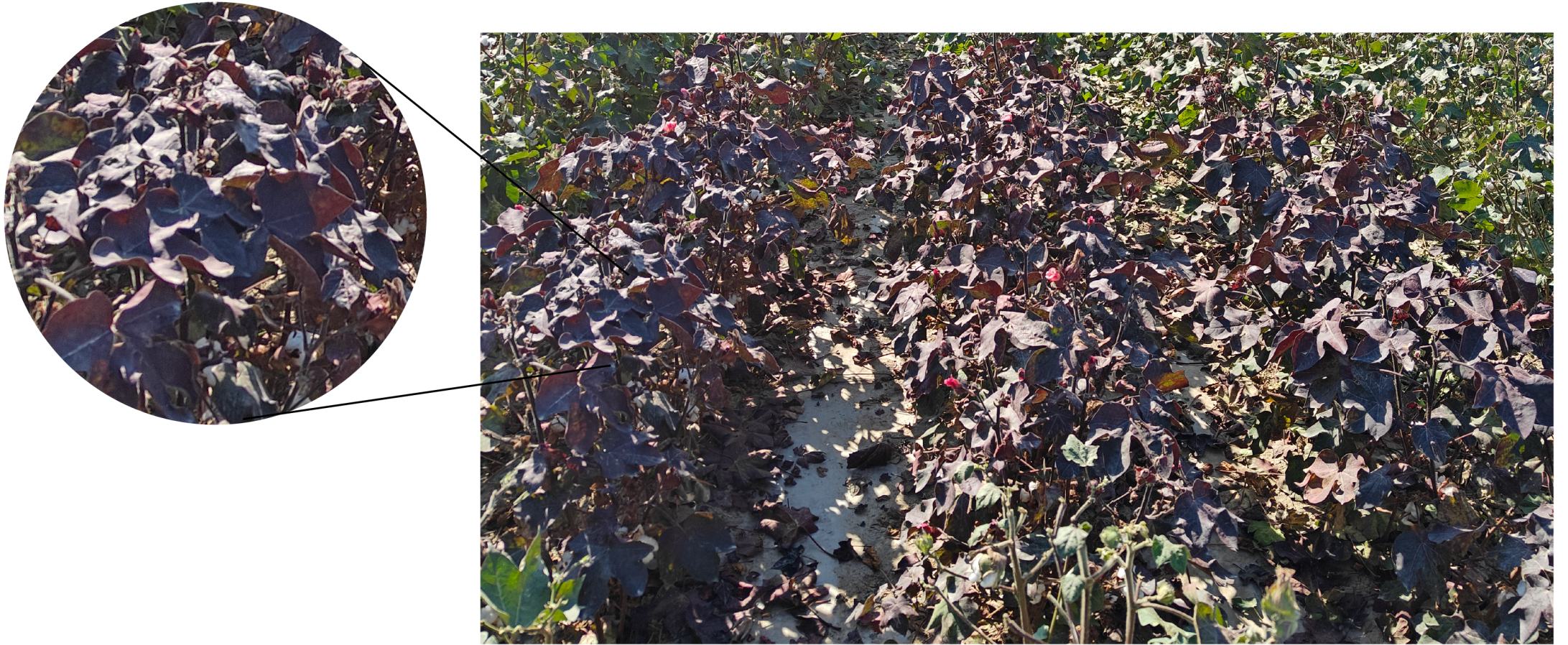

**Supplementary Figure 1.** Field phenotype of red leaf cotton 20 days after rewetting.

Supplement: Supplementary file 1 [file DataSheet1.pdf]
