# Supplementary material for: Transcriptomics and non-targeted metabolomics reveal the mechanisms of leaf color changes in red-leaf cotton under drought stress and rewatering
Source: Front Plant Sci. 2026 Feb 16;17:1766818. doi: 10.3389/fpls.2026.1766818 (PMC12951637; doi:10.3389/fpls.2026.1766818)
Supplement: Supplementary file 2 [file DataSheet2.pdf]

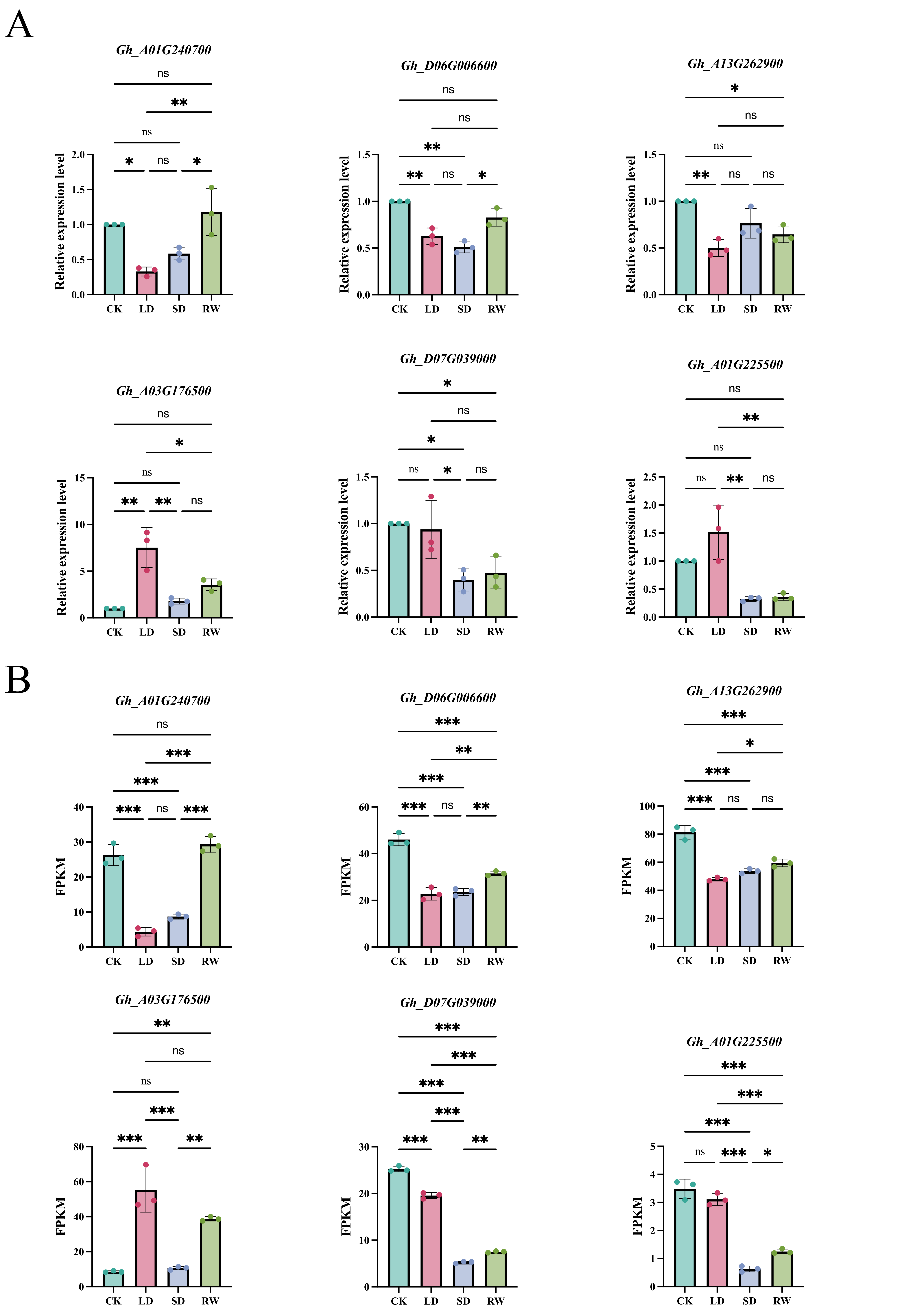

Supplementary Figure 2. Expression validation of candidate genes under drought stress and rewatering. (A) qRT-PCR analysis: Quantitative reverse transcription PCR (qRT-PCR) was used to analyze the expression patterns of candidate genes under different drought stresses and rewatering conditions. Error bars represent the standard error (SE) of three biological replicates. Significance levels: * $p < 0.05$, ** $p < 0.01$, *** $p < 0.001$ (Student's t-test). (B) RNA-seq validation: Transcript abundance of candidate genes was quantified by fragments per million bases (FPKM).
